# *catena*-Poly[[bis­(1-methyl­imidazole-κ*N* ^3^)zinc(II)]-μ-isophthalato-κ^2^ *O* ^1^:*O* ^3^]

**DOI:** 10.1107/S160053680803078X

**Published:** 2008-09-27

**Authors:** Juan Zhao

**Affiliations:** a College of Mechanical Engineering, Qingdao Technological University, Qingdao 266033, People’s Republic of China

## Abstract

In the solid state, the title compound, [Zn(C_8_H_4_O_4_)(C_4_H_6_N_2_)_2_]_*n*_, exhibits the existence of polymeric zigzag chains extending along the *a* axis. Each Zn^II^ ion is coordinated by two N atoms [Zn—N = 1.996 (6) and 2.032 (5) Å] and two O atoms [Zn—O = 1.930 (4) and 1.976 (4) Å] in a distorted tetra­hedral geometry. Weak C—H⋯O inter­actions contribute to the crystal packing stability.

## Related literature

In the related zinc compound [Zn(isophthalato)(1-*H*-imidazole)_2_] (Yang *et al.*, 2002 ▶), the Zn^II^ ions also have a distorted tetra­hedral environment.
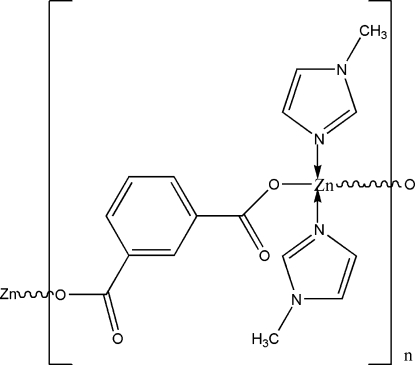


## Experimental

### 

#### Crystal data

[Zn(C_8_H_4_O_4_)(C_4_H_6_N_2_)_2_]
*M* *_r_* = 393.72
Orthorhombic, 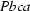

*a* = 9.6820 (19) Å
*b* = 13.224 (3) Å
*c* = 26.983 (5) Å
*V* = 3454.8 (12) Å^3^
*Z* = 8
Mo *K*α radiation
μ = 1.45 mm^−1^
*T* = 293 (2) K
0.20 × 0.10 × 0.10 mm

#### Data collection

Enraf–Nonius CAD-4 diffractometer
Absorption correction: none
3091 measured reflections
2967 independent reflections
2041 reflections with *I* > 2σ(*I*)
*R*_int_ = 0.035
3 standard reflections every 100 reflections intensity decay: none

#### Refinement

*R*[*F* ^2^ > 2σ(*F* ^2^)] = 0.068
*wR*(*F* ^2^) = 0.207
*S* = 1.05
2967 reflections
220 parameters
40 restraints
H-atom parameters constrained
Δρ_max_ = 0.50 e Å^−3^
Δρ_min_ = −0.61 e Å^−3^

### 

Data collection: *CAD-4 Software* (Enraf–Nonius, 1989 ▶); cell refinement: *CAD-4 Software*; data reduction: *NRCVAX* (Gabe *et al.*, 1989 ▶); program(s) used to solve structure: *SHELXS97* (Sheldrick, 2008 ▶); program(s) used to refine structure: *SHELXL97* (Sheldrick, 2008 ▶); molecular graphics: *SHELXTL/PC* (Sheldrick, 2008 ▶); software used to prepare material for publication: *WinGX* (Farrugia, 1999 ▶).

## Supplementary Material

Crystal structure: contains datablocks global, I. DOI: 10.1107/S160053680803078X/cv2451sup1.cif

Structure factors: contains datablocks I. DOI: 10.1107/S160053680803078X/cv2451Isup2.hkl

Additional supplementary materials:  crystallographic information; 3D view; checkCIF report

## Figures and Tables

**Table 1:** Hydrogen-bond geometry (Å, °)

*D*—H⋯*A*	*D*—H	H⋯*A*	*D*⋯*A*	*D*—H⋯*A*
C1—H1*A*⋯O3^i^	0.96	2.55	3.423 (10)	150
C4—H4*B*⋯O3^i^	0.93	2.31	3.150 (9)	150
C11—H11*A*⋯O2^ii^	0.93	2.54	3.457 (8)	171

Symmetry codes: (i) 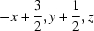; (ii) 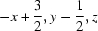.

**Fig. 1.**
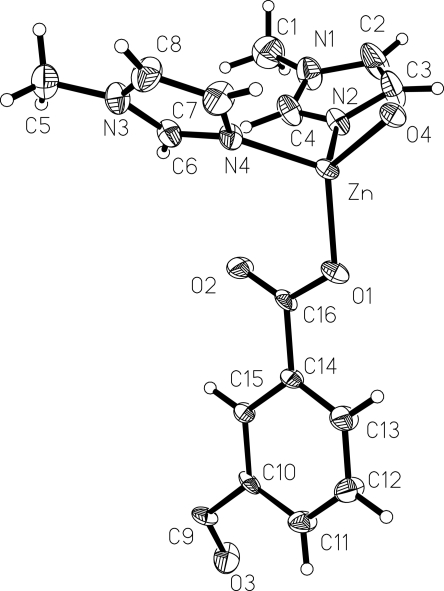
A portion of the polymeric chain in (I) showing the atomic numbering and 30% probability displacement ellipsoids.

## supplementary crystallographic information

### Comment

In the title compound, (I) (Fig. 1), the zinc(II) centers are bridged by carboxylate groups of isophthalate ligands. Each Zn^II^ ion is coordinated by two N [Zn—N2 = 1.996 (6) Å, Zn—N4 = 2.032 (5) Å] and two O [Zn—O1 = 1.930 (4) Å, Zn—O4 = 1.976 (4) Å] atoms in a distorted tetrahedral geometry. All these values agree well with those observed in [Zn(isophthalato)(1-*H*-imidazole)_2_] (Yang *et al.*, 2002). Each isophthalate dianion in (I) acts as a bidentate ligand to bridge two Zn^II^ atoms through two monodentate carboxylate groups, building a zigzag polymeric chain along the *a* axis. The metal–metal distance across each polymer backbone is 9.682 (7) Å.

In the crystal, weak C—H···O interactions contribute to the crystal packing stability. In the corresponding zinc compound [Zn(isophthalato)(1-*H*-imidazole)_2_] (Yang *et al.*, 2002), the Zn^II^ ions have a distorted tetrahedral environment.

### Experimental

The reaction of ZnCl_2_(0.68 g, 5 mmol) with isophthalic acid (0.83 g, 5 mmol) in an aqueous-alcohol (3:1) solution (40 ml) at 363 K for 30 minutes produced a blue solution, to which 1-methylimidazole (0.82 g, 10 mmol) was added. The reaction solution was kept at room temperature after stirring for an hour at 333 K. Colourless crystals were obtained after a few days.

### Refinement

H atoms were positioned geometrically (C—H = 0.93 or 0.96 Å) and allowed to ride on their parent atoms with *U*_iso_(H) = 1.2 or 1.5 times *U*_eq_(C).

### Crystal data

**Table:**

[Zn(C_8_H_4_O_4_)(C_4_H_6_N_2_)_2_]	*F*(000) = 1616
*M**_r_* = 393.72	*D*_x_ = 1.514 Mg m^−^^3^
Orthorhombic, *P**b**c**a*	Mo *K*α radiation, λ = 0.71073 Å
Hall symbol: -P 2ac 2ab	Cell parameters from 25 reflections
*a* = 9.6820 (19) Å	θ = 10–14°
*b* = 13.224 (3) Å	µ = 1.45 mm^−^^1^
*c* = 26.983 (5) Å	*T* = 293 K
*V* = 3454.8 (12) Å^3^	Block, colourless
*Z* = 8	0.20 × 0.10 × 0.10 mm

### Data collection

**Table:**

Enraf–Nonius CAD-4 diffractometer	*R*_int_ = 0.036
Radiation source: fine-focus sealed tube	θ_max_ = 25.2°, θ_min_ = 1.5°
graphite	*h* = 0→11
ω scans	*k* = 0→15
3091 measured reflections	*l* = 0→32
2967 independent reflections	3 standard reflections every 100 reflections
2041 reflections with *I* > 2σ(*I*)	intensity decay: none

### Refinement

**Table:**

Refinement on *F*^2^	Primary atom site location: structure-invariant direct methods
Least-squares matrix: full	Secondary atom site location: difference Fourier map
*R*[*F*^2^ > 2σ(*F*^2^)] = 0.068	Hydrogen site location: inferred from neighbouring sites
*wR*(*F*^2^) = 0.207	H-atom parameters constrained
*S* = 1.05	*w* = 1/[σ^2^(*F*_o_^2^) + (0.1255*P*)^2^] where *P* = (*F*_o_^2^ + 2*F*_c_^2^)/3
2967 reflections	(Δ/σ)_max_ < 0.001
220 parameters	Δρ_max_ = 0.50 e Å^−^^3^
40 restraints	Δρ_min_ = −0.61 e Å^−^^3^

### Special details

**Table:**

Geometry. All e.s.d.'s (except the e.s.d. in the dihedral angle between two l.s. planes) are estimated using the full covariance matrix. The cell e.s.d.'s are taken into account individually in the estimation of e.s.d.'s in distances, angles and torsion angles; correlations between e.s.d.'s in cell parameters are only used when they are defined by crystal symmetry. An approximate (isotropic) treatment of cell e.s.d.'s is used for estimating e.s.d.'s involving l.s. planes.
Refinement. Refinement of *F*^2^ against ALL reflections. The weighted *R*-factor *wR* and goodness of fit *S* are based on *F*^2^, conventional *R*-factors *R* are based on *F*, with *F* set to zero for negative *F*^2^. The threshold expression of *F*^2^ > σ(*F*^2^) is used only for calculating *R*-factors(gt) *etc*. and is not relevant to the choice of reflections for refinement. *R*-factors based on *F*^2^ are statistically about twice as large as those based on *F*, and *R*- factors based on ALL data will be even larger.

### Fractional atomic coordinates and isotropic or equivalent isotropic displacement parameters (Å^2^)

**Table:**

	*x*	*y*	*z*	*U*_iso_*/*U*_eq_	
Zn	0.18405 (6)	0.12667 (5)	0.12048 (3)	0.0338 (3)	
N1	0.1883 (6)	0.3348 (5)	0.2326 (2)	0.0594 (17)	
N2	0.1689 (5)	0.2071 (4)	0.1829 (2)	0.0434 (13)	
N3	0.2381 (6)	0.3608 (4)	0.0199 (2)	0.0461 (14)	
N4	0.1948 (5)	0.2244 (4)	0.06251 (18)	0.0358 (12)	
O1	0.3326 (4)	0.0294 (4)	0.11870 (16)	0.0460 (12)	
O2	0.4748 (4)	0.1603 (3)	0.12273 (18)	0.0510 (13)	
O3	1.0442 (4)	−0.0567 (4)	0.1520 (2)	0.0680 (16)	
O4	−0.0041 (4)	0.0763 (3)	0.10656 (16)	0.0440 (11)	
C1	0.2355 (10)	0.4326 (6)	0.2511 (3)	0.081	
H1A	0.3017	0.4605	0.2284	0.122*	
H1B	0.2777	0.4239	0.2830	0.122*	
H1C	0.1581	0.4776	0.2539	0.122*	
C2	0.0944 (8)	0.2711 (7)	0.2534 (3)	0.079 (3)	
H2A	0.0483	0.2798	0.2833	0.094*	
C3	0.0821 (8)	0.1945 (7)	0.2223 (3)	0.071 (2)	
H3A	0.0229	0.1399	0.2267	0.086*	
C4	0.2306 (8)	0.2910 (6)	0.1911 (2)	0.0523 (18)	
H4B	0.2976	0.3181	0.1703	0.063*	
C5	0.2982 (8)	0.4589 (5)	0.0065 (3)	0.060 (2)	
H5A	0.3727	0.4745	0.0288	0.089*	
H5B	0.2287	0.5104	0.0089	0.089*	
H5C	0.3326	0.4560	−0.0268	0.089*	
C6	0.2723 (6)	0.3054 (5)	0.0588 (2)	0.0408 (15)	
H6A	0.3427	0.3218	0.0809	0.049*	
C7	0.1029 (8)	0.2310 (5)	0.0239 (3)	0.0568 (19)	
H7A	0.0326	0.1849	0.0175	0.068*	
C8	0.1294 (8)	0.3137 (5)	−0.0030 (3)	0.059 (2)	
H8A	0.0835	0.3349	−0.0315	0.070*	
C9	0.9641 (6)	−0.0064 (5)	0.1281 (2)	0.0361 (14)	
C10	0.8172 (5)	−0.0403 (5)	0.1219 (2)	0.0328 (14)	
C11	0.7891 (7)	−0.1438 (5)	0.1187 (2)	0.0436 (16)	
H11A	0.8608	−0.1907	0.1190	0.052*	
C12	0.6544 (7)	−0.1755 (5)	0.1151 (3)	0.060 (2)	
H12A	0.6356	−0.2443	0.1126	0.072*	
C13	0.5473 (7)	−0.1080 (5)	0.1151 (3)	0.0510 (18)	
H13A	0.4572	−0.1318	0.1121	0.061*	
C14	0.5702 (5)	−0.0054 (4)	0.1195 (2)	0.0301 (12)	
C15	0.7082 (5)	0.0286 (4)	0.1222 (2)	0.0321 (13)	
H15A	0.7265	0.0975	0.1242	0.039*	
C16	0.4545 (6)	0.0702 (5)	0.1210 (2)	0.0340 (14)	

### Atomic displacement parameters (Å^2^)

**Table:**

	*U*^11^	*U*^22^	*U*^33^	*U*^12^	*U*^13^	*U*^23^
Zn	0.0166 (4)	0.0401 (4)	0.0446 (5)	0.0005 (3)	−0.0007 (3)	0.0023 (3)
N1	0.067 (4)	0.058 (3)	0.053 (3)	−0.018 (3)	0.016 (3)	−0.013 (3)
N2	0.032 (3)	0.056 (3)	0.043 (3)	−0.008 (2)	0.006 (2)	0.001 (2)
N3	0.051 (3)	0.052 (3)	0.035 (3)	−0.008 (3)	0.003 (3)	0.003 (3)
N4	0.024 (2)	0.043 (3)	0.041 (3)	−0.009 (2)	−0.009 (2)	0.006 (2)
O1	0.019 (2)	0.057 (3)	0.062 (3)	0.004 (2)	0.0020 (19)	0.003 (2)
O2	0.021 (2)	0.036 (2)	0.096 (4)	−0.0011 (19)	−0.001 (2)	0.004 (2)
O3	0.023 (2)	0.075 (3)	0.106 (4)	−0.001 (2)	−0.013 (3)	0.041 (3)
O4	0.020 (2)	0.049 (3)	0.063 (3)	−0.0021 (19)	−0.004 (2)	0.008 (2)
C1	0.081	0.081	0.081	0.000	0.000	0.000
C2	0.068 (5)	0.100 (6)	0.068 (5)	−0.024 (4)	0.038 (4)	−0.019 (4)
C3	0.064 (5)	0.081 (5)	0.069 (5)	−0.030 (4)	0.031 (4)	−0.006 (4)
C4	0.052 (4)	0.071 (4)	0.033 (3)	−0.010 (3)	0.015 (3)	−0.005 (3)
C5	0.069 (5)	0.054 (4)	0.056 (5)	−0.010 (4)	0.008 (4)	0.004 (4)
C6	0.034 (3)	0.051 (4)	0.037 (4)	−0.006 (3)	0.001 (3)	0.003 (3)
C7	0.053 (4)	0.054 (4)	0.063 (5)	−0.009 (4)	−0.017 (4)	0.005 (4)
C8	0.061 (5)	0.060 (5)	0.054 (5)	−0.004 (4)	−0.017 (4)	0.017 (4)
C9	0.017 (3)	0.046 (3)	0.045 (4)	0.006 (3)	0.000 (3)	−0.007 (3)
C10	0.011 (3)	0.054 (4)	0.033 (3)	−0.002 (2)	0.002 (2)	0.005 (3)
C11	0.026 (3)	0.041 (4)	0.064 (4)	0.005 (3)	−0.006 (3)	−0.007 (3)
C12	0.029 (4)	0.031 (3)	0.119 (7)	−0.006 (3)	−0.005 (4)	−0.006 (4)
C13	0.024 (3)	0.042 (4)	0.087 (5)	−0.005 (3)	−0.002 (3)	−0.007 (3)
C14	0.015 (3)	0.037 (3)	0.038 (3)	0.002 (2)	−0.004 (2)	0.001 (3)
C15	0.017 (3)	0.034 (3)	0.046 (4)	−0.001 (2)	−0.002 (2)	0.005 (3)
C16	0.012 (3)	0.049 (4)	0.041 (3)	0.001 (3)	0.001 (2)	0.008 (3)

### Geometric parameters (Å, °)

**Table:**

Zn—O1	1.930 (4)	C3—H3A	0.9300
Zn—O4	1.976 (4)	C4—H4B	0.9300
Zn—N2	1.996 (6)	C5—H5A	0.9600
Zn—N4	2.032 (5)	C5—H5B	0.9600
N1—C4	1.327 (8)	C5—H5C	0.9600
N1—C2	1.360 (9)	C6—H6A	0.9300
N1—C1	1.459 (10)	C7—C8	1.339 (9)
N2—C4	1.279 (8)	C7—H7A	0.9300
N2—C3	1.367 (8)	C8—H8A	0.9300
N3—C6	1.322 (8)	C9—O4^ii^	1.277 (8)
N3—C8	1.371 (9)	C9—C10	1.501 (7)
N3—C5	1.468 (8)	C10—C15	1.395 (8)
N4—C6	1.311 (7)	C10—C11	1.399 (9)
N4—C7	1.372 (8)	C11—C12	1.373 (8)
O1—C16	1.299 (7)	C11—H11A	0.9300
O2—C16	1.209 (8)	C12—C13	1.368 (9)
O3—C9	1.207 (7)	C12—H12A	0.9300
O4—C9^i^	1.277 (8)	C13—C14	1.381 (8)
C1—H1A	0.9600	C13—H13A	0.9300
C1—H1B	0.9600	C14—C15	1.411 (7)
C1—H1C	0.9600	C14—C16	1.502 (8)
C2—C3	1.319 (11)	C15—H15A	0.9300
C2—H2A	0.9300		
			
O1—Zn—O4	117.23 (19)	H5A—C5—H5B	109.5
O1—Zn—N2	115.5 (2)	N3—C5—H5C	109.5
O4—Zn—N2	105.80 (19)	H5A—C5—H5C	109.5
O1—Zn—N4	111.51 (19)	H5B—C5—H5C	109.5
O4—Zn—N4	96.63 (18)	N4—C6—N3	111.7 (6)
N2—Zn—N4	108.3 (2)	N4—C6—H6A	124.2
C4—N1—C2	106.5 (6)	N3—C6—H6A	124.2
C4—N1—C1	125.3 (7)	C8—C7—N4	109.9 (6)
C2—N1—C1	128.2 (7)	C8—C7—H7A	125.1
C4—N2—C3	104.9 (6)	N4—C7—H7A	125.1
C4—N2—Zn	125.0 (5)	C7—C8—N3	105.8 (6)
C3—N2—Zn	129.6 (5)	C7—C8—H8A	127.1
C6—N3—C8	107.4 (5)	N3—C8—H8A	127.1
C6—N3—C5	125.9 (6)	O3—C9—O4^ii^	124.1 (6)
C8—N3—C5	126.5 (6)	O3—C9—C10	120.2 (6)
C6—N4—C7	105.2 (5)	O4^ii^—C9—C10	115.7 (5)
C6—N4—Zn	127.4 (4)	C15—C10—C11	119.5 (5)
C7—N4—Zn	126.2 (4)	C15—C10—C9	121.5 (6)
C16—O1—Zn	113.5 (4)	C11—C10—C9	118.9 (5)
C9^i^—O4—Zn	115.1 (4)	C12—C11—C10	119.2 (6)
N1—C1—H1A	109.5	C12—C11—H11A	120.4
N1—C1—H1B	109.5	C10—C11—H11A	120.4
H1A—C1—H1B	109.5	C13—C12—C11	121.4 (6)
N1—C1—H1C	109.5	C13—C12—H12A	119.3
H1A—C1—H1C	109.5	C11—C12—H12A	119.3
H1B—C1—H1C	109.5	C12—C13—C14	121.2 (6)
C3—C2—N1	105.9 (7)	C12—C13—H13A	119.4
C3—C2—H2A	127.0	C14—C13—H13A	119.4
N1—C2—H2A	127.0	C13—C14—C15	118.0 (5)
C2—C3—N2	110.2 (7)	C13—C14—C16	122.4 (5)
C2—C3—H3A	124.9	C15—C14—C16	119.5 (5)
N2—C3—H3A	124.9	C10—C15—C14	120.5 (5)
N2—C4—N1	112.4 (6)	C10—C15—H15A	119.7
N2—C4—H4B	123.8	C14—C15—H15A	119.7
N1—C4—H4B	123.8	O2—C16—O1	124.0 (6)
N3—C5—H5A	109.5	O2—C16—C14	122.4 (5)
N3—C5—H5B	109.5	O1—C16—C14	113.6 (5)
			
O1—Zn—N2—C4	89.0 (6)	Zn—N4—C6—N3	−169.7 (4)
O4—Zn—N2—C4	−139.6 (6)	C8—N3—C6—N4	0.9 (8)
N4—Zn—N2—C4	−36.9 (6)	C5—N3—C6—N4	176.2 (6)
O1—Zn—N2—C3	−100.1 (7)	C6—N4—C7—C8	1.9 (8)
O4—Zn—N2—C3	31.4 (7)	Zn—N4—C7—C8	170.1 (5)
N4—Zn—N2—C3	134.1 (7)	N4—C7—C8—N3	−1.4 (9)
O1—Zn—N4—C6	−81.7 (5)	C6—N3—C8—C7	0.3 (9)
O4—Zn—N4—C6	155.6 (5)	C5—N3—C8—C7	−175.0 (6)
N2—Zn—N4—C6	46.5 (6)	O3—C9—C10—C15	141.9 (7)
O1—Zn—N4—C7	112.7 (6)	O4^ii^—C9—C10—C15	−38.6 (9)
O4—Zn—N4—C7	−10.0 (6)	O3—C9—C10—C11	−34.3 (9)
N2—Zn—N4—C7	−119.1 (6)	O4^ii^—C9—C10—C11	145.2 (6)
O4—Zn—O1—C16	170.5 (4)	C15—C10—C11—C12	1.0 (10)
N2—Zn—O1—C16	−63.7 (4)	C9—C10—C11—C12	177.3 (6)
N4—Zn—O1—C16	60.4 (4)	C10—C11—C12—C13	−0.7 (12)
O1—Zn—O4—C9^i^	44.5 (5)	C11—C12—C13—C14	−1.0 (12)
N2—Zn—O4—C9^i^	−86.0 (4)	C12—C13—C14—C15	2.4 (11)
N4—Zn—O4—C9^i^	162.9 (4)	C12—C13—C14—C16	−178.4 (6)
C4—N1—C2—C3	−2.1 (10)	C11—C10—C15—C14	0.3 (9)
C1—N1—C2—C3	177.6 (8)	C9—C10—C15—C14	−175.9 (5)
N1—C2—C3—N2	1.5 (11)	C13—C14—C15—C10	−2.0 (10)
C4—N2—C3—C2	−0.2 (10)	C16—C14—C15—C10	178.7 (5)
Zn—N2—C3—C2	−172.6 (6)	Zn—O1—C16—O2	−1.8 (8)
C3—N2—C4—N1	−1.2 (9)	Zn—O1—C16—C14	−180.0 (4)
Zn—N2—C4—N1	171.6 (5)	C13—C14—C16—O2	−176.8 (7)
C2—N1—C4—N2	2.1 (9)	C15—C14—C16—O2	2.4 (9)
C1—N1—C4—N2	−177.6 (7)	C13—C14—C16—O1	1.4 (8)
C7—N4—C6—N3	−1.7 (7)	C15—C14—C16—O1	−179.4 (5)

Symmetry codes: (i) *x*−1, *y*, *z*; (ii) *x*+1, *y*, *z*.

### Hydrogen-bond geometry (Å, °)

**Table:**

*D*—H···*A*	*D*—H	H···*A*	*D*···*A*	*D*—H···*A*
C1—H1A···O3^iii^	0.96	2.55	3.423 (10)	150
C4—H4B···O3^iii^	0.93	2.31	3.150 (9)	150
C11—H11A···O2^iv^	0.93	2.54	3.457 (8)	171

Symmetry codes: (iii) −*x*+3/2, *y*+1/2, *z*; (iv) −*x*+3/2, *y*−1/2, *z*.

## References

[bb1] Enraf–Nonius (1989). *CAD-4 Software* Enraf–Nonius, Delft, The Netherlands.

[bb2] Farrugia, L. J. (1999). *J. Appl. Cryst.* **32**, 837–838.

[bb3] Gabe, E. J., Le Page, Y., Charland, J.-P., Lee, F. L. & White, P. S. (1989). *J. Appl. Cryst.* **22**, 384–387.

[bb4] Sheldrick, G. M. (2008). *Acta Cryst.* A**64**, 112–122. 10.1107/S0108767307043930 18156677

[bb5] Yang, J., Zheng, S., Tao, J., Liu, G. & Chen, X. (2002). *Aust. J. Chem.* **55**, 741–744.

